# Investigation of Griffithsin's Interactions with Human Cells Confirms Its Outstanding Safety and Efficacy Profile as a Microbicide Candidate

**DOI:** 10.1371/journal.pone.0022635

**Published:** 2011-08-02

**Authors:** Joseph Calvin Kouokam, Dana Huskens, Dominique Schols, Andrew Johannemann, Shonna K. Riedell, Wendye Walter, Janice M. Walker, Nobuyuki Matoba, Barry R. O'Keefe, Kenneth E. Palmer

**Affiliations:** 1 Owensboro Cancer Research Program, James Graham Brown Cancer Center and Department of Pharmacology and Toxicology, University of Louisville School of Medicine, Louisville, Kentucky, United States of America; 2 Rega Institute for Medical Research, K.U. Leuven, Leuven, Belgium; 3 Molecular Targets Laboratory, Center for Cancer Research, National Cancer Institute, Frederick, Maryland, United States of America; Center for Disease Control and Prevention, United States of America

## Abstract

Many natural product-derived lectins such as the red algal lectin griffithsin (GRFT) have potent *in vitro* activity against viruses that display dense clusters of oligomannose N-linked glycans (NLG) on their surface envelope glycoproteins. However, since oligomannose NLG are also found on some host proteins it is possible that treatment with antiviral lectins may trigger undesirable side effects. For other antiviral lectins such as concanavalin A, banana lectin and cyanovirin-N (CV-N), interactions between the lectin and as yet undescribed cellular moieties have been reported to induce undesirable side effects including secretion of inflammatory cytokines and activation of host T-cells. We show that GRFT, unlike CV-N, binds the surface of human epithelial and peripheral blood mononuclear cells (PBMC) through an exclusively oligosaccharide-dependent interaction. In contrast to several other antiviral lectins however, GRFT treatment induces only minimal changes in secretion of inflammatory cytokines and chemokines by epithelial cells or human PBMC, has no measureable effect on cell viability and does not significantly upregulate markers of T-cell activation. In addition, GRFT appears to retain antiviral activity once bound to the surface of PBMC. Finally, RNA microarray studies show that, while CV-N and ConA regulate expression of a multitude of cellular genes, GRFT treatment effects only minimal alterations in the gene expression profile of a human ectocervical cell line. These studies indicate that GRFT has an outstanding safety profile with little evidence of induced toxicity, T-cell activation or deleterious immunological consequence, unique attributes for a natural product-derived lectin.

## Introduction

HIV-1 is the prototype example of a virus that utilizes an oligomannose-rich “glycan shield” to occlude functionally important domains of the envelope glycoproteins from antibodies, and evade the immune response [1], [2]. Recently Doores *et al.* [3] showed that previous measurement of the proportion of oligomannose NLG relative to complex NLG on recombinant HIV envelope glycoproteins underestimated the representation of oligomannose NLG on the native envelope spikes of HIV-1, which appear to display NLG that are almost exclusively mannose-terminal Man_5–9_-GlcNAc_2_ structures. It is likely that limited access to the high density of NLG presented on the HIV-1 trimeric glycoprotein spike by Golgi and endoplasmic reticulum (ER) α1→2 mannosidases results in an atypical preponderance of oligomannose glycans rather than complex NLG on HIV-1 surface glycoproteins [3], [4], [5]. Given that Man_5–9_-GlcNAc_2_ structures are present on less than 4% of the normal human N-glycome [6], [7], dense clusters of oligomannose NLG appear to be a feature specific to viral envelope glycoproteins, particularly those of HIV-1 and other immunodeficiency lentiviruses [3]. Consequently, clusters of oligomannose NLG may be attractive molecular targets for antiviral drugs and vaccines that act to interrupt HIV-1 infection of target cells by: (i) binding on the virus envelope and thereby interfering with the structural transitions involved in receptor and co-receptor docking and virus entry into T-cells and (ii) blocking access to viral envelope oligomannose NLG targeted by C-type lectin receptors DC-SIGN and MMR on dendritic cells and macrophages [5], [8], [9].

It has long been known that a variety of oligomannose-specific lectins have potent *in vitro* HIV-1 inhibitory activities, and therefore have been proposed as microbicide candidates for topical prophylaxis of HIV-1 infection, and as potential therapeutics [8], [9]. However many lectins possess lymphocyte mitogenic activities incompatible with their use as pharmaceuticals, and some are known human and animal toxins, although the pharmacological basis for their toxicity is poorly characterized [10], [11]. The antiviral potency of lectins has been correlated to their capacity to bind multiple glycans simultaneously, often facilitated by their ability to form dimers and higher order multimers [12], [13], [14].

The most extensively characterized antiviral lectin is CV-N, a small protein that exists in both monomeric and homodimeric configurations and has exceptionally potent anti-HIV activity, in the low nanomolar range [15]. CV-N targets the Manα1→2Man terminating glycans displayed in the Man_6–9_GlcNAc_2_ structures on the surface of many viral envelope glycoproteins. Each monomer of CV-N has the capacity to bind two oligomannose structures [16]. When formulated into a carboxyethylcellulose gel matrix, CV-N provided almost complete protection against a single high dose intrarectal or intravaginal challenge with a pathogenic simian-human immunodeficiency virus (SHIV) [17], [18]. However, subsequent *in vitro* toxicity studies have raised concerns about the safety of CV-N based microbicides, finding that *in vitro*, CV-N has the capacity to promote secretion of pro-inflammatory cytokines and chemokines from human peripheral blood mononuclear cells (PBMC), activate quiescent CD4^+^ T-cells, and promote T-cell proliferation [19], [20], [21], [22]. Similar results were also reported for other lectins such as microvirin (MVN) (17) and concanavalin A (Con A) (18). It should be noted that the toxicities of CV-N were much milder in treated cervical explants in comparison with PBMC [20], [21]. The possible pathogenic consequences associated with these off-target activities have raised concerns about all other members of the natural product-based class of antiviral lectins [19], [20], [21].

GRFT has the most potent and broad spectrum HIV-1 inhibitory activity yet described for any antiviral lectin [15], [23], [24], [25]. It is a 25 kDa domain-swapped homodimer, with the first 16 amino acids of each 12.7 kDa monomer completing the β-prism fold of the other [26]. The homodimer has six carbohydrate binding pockets, 3 located at each of the opposite ends of the double-prism homodimer. Atomic resolution crystal structures of an engineered monomeric GRFT showed that each monomer can bind to two different nonamannoside molecules through all three carbohydrate binding sites [13], [26]. The antiviral activity of monomeric GRFT is substantially lower than that of the homodimeric form, confirming that the GRFT potency is dependent on its ability to bind multiple oligomannose structures simultaneously, with strong avidity [13]. We showed recently that GRFT causes no mitogenic stimulation of PBMC exposed to the drug [23]. GRFT is fully active in the presence of macaque vaginal secretions [25], and was shown to have a good safety profile in the rabbit vaginal irritation model, the Gold Standard preclinical safety test for vaginal products [23]. Moreover, treatment of human cervical explants with GRFT induced minimal alterations in the expression profile of a panel of proinflammatory chemokines and cytokines. GRFT also strongly inhibited HIV-1 infection of the cervical explants, and dissemination of HIV-1 infection from cells resident in the explants to donor T-cells [23]. In the present study, we performed a comprehensive set of experiments to interrogate the molecular response of cultured human cervico-vaginal cells and PBMC to GRFT exposure. Our investigations employed comparisons between the biological activities of GRFT, which binds mannose-terminal Man_5–9_-GlcNAc_2_, with other lectins of well-defined carbohydrate binding specificity: (1) CV-N, which binds Manα1→2Man terminating glycans on Man_6–9_-GlcNAc_2_ structures; (2) phytohemagglutinin A (PHA), targeting D-galactose and N-acetyl-D-galactosamine on gycan structures; (3) ConA, specific for terminal (tri) mannose on high mannose glycans and (4) Pokeweed agglutinin (PKM), which binds N-acetylglucosamine. Our studies reveal clear distinctions in biological and toxicological properties of these lectins, and confirm GRFT's superior safety profile for use as a topical microbicide.

## Results

### GRFT and CV-N binding to human cervical epithelium, cultured human cervicovaginal cells, and PBMC

**Figure 1:**
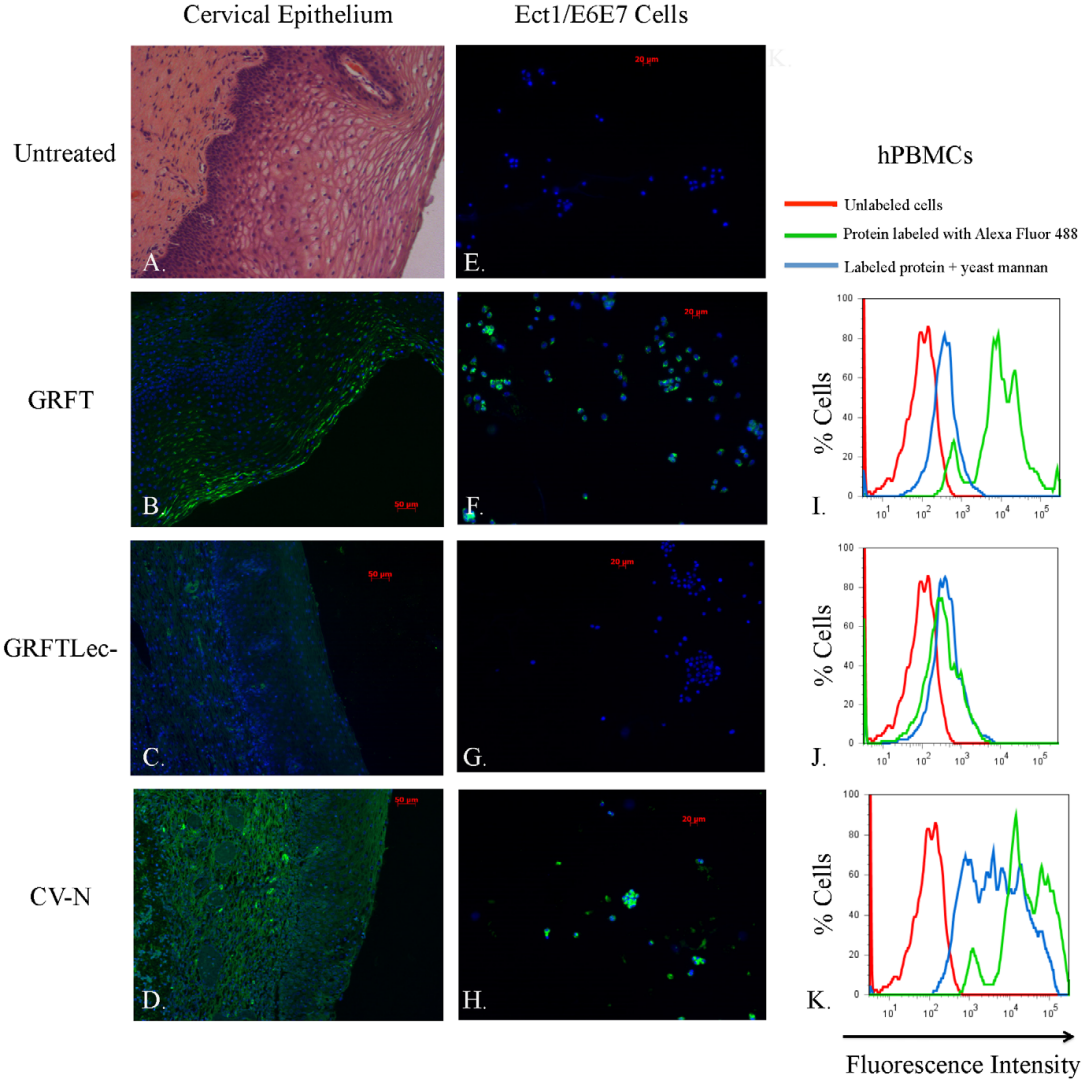
Analysis of binding specificity of GRFT in comparison with GRFT^lec-^ and CV-N. In the first column we depict fluorescence micrographs that show binding of AlexaFluor 488-labeled GRFT (B.), GRFT^Lec-^ (C.) and CV-N (D.) to paraffin-embedded cervical tissue sections from a 21-year old female. In (A.) we show a hematoxilin and eosin-stained light micrograph of cervical epithelial tissue, showing the general micro-anatomy of the cervical epithelium. In the second column, we show fluorescent micrographs of cultured human ectocervical cell line Ec1/E6E7 untreated (E.), and treated with AlexaFluor 488-labeled GRFT (F.), GRFTLec- (G.) and CV-N (H.). The third column shows histograms that depict flow cytometric analysis of hPMBCs treated with fluorescently labeled GRFT (I.), GRFT^Lec-^ (J.) and CV-N (K.). The X-axis shows increasing fluorescence intensity from left to right, the Y-axis shows percentage of labeled cells in the sample.

We used paraffin-embedded cervical epithelial sections from a 21-year old donor to evaluate the binding pattern of fluorescently labeled GRFT and CV-N to human mucosal epithelia. GRFT^Lec-^, a mutant form of GRFT where we eliminated the lectin activity through mutation of all six mannose binding sites, was used as a control to help distinguish binding mediated by the GRFT carbohydrate binding pockets versus binding associated with other GRFT structures. The light micrograph in Figure 1A shows an H&E stained cervical tissue section to orient the observer to the microanatomy of the human cervical epithelium. Different layers of the squamous epithelium starting at the basement membrane (basal, parabasal, intermediate, superficial) are evident; cervical connective tissue or stroma is beneath the basement membrane. Tissues incubated with labeled GRFT, GRFT^Lec-^ and CV-N are shown in Fig. 1B, C and D, respectively. Minimal fluorescence seen in tissues exposed to GRFT^Lec-^ (Fig. 1C) compared with GRFT-stained tissues (Fig. 1B) confirmed that the binding of GRFT to the outermost layer of the squamous epithelium was via its carbohydrate binding activity. There were distinct differences evident in the binding pattern of GRFT (Fig. 1C) relative to CV-N (Fig. 1D), which bound far more extensively than GRFT throughout all layers of the squamous epithelium, basement membrane and underlying stromal tissue. Additional fluorescence micrographs are provided in Fig. S1. In cultured cervicovaginal epithelial cells we also observed binding of both GRFT and CV-N, but not GRFT^Lec-^ to Ect1/E6E7 cells (compare Fig. 1E through H), as well as End1/E6E7 and VK2/E6E7 cells (data not shown). We used flow cytometry to evaluate binding of fluorescently labeled GRFT, GRFT^Lec-^ and CV-N to human PBMC. Clear shifts in fluorescence intensity show that GRFT (Fig. 1 I) and CV-N (Fig. 1K) efficiently bind the surface of human PBMC relative to GRFT^Lec-^ (Fig. 1J), for which we observed only minimal binding. Binding of GRFT to the surface of PBMC was significantly reduced when occluding the glycan binding pockets by pre-incubation with yeast mannan (Fig. 1I). Interestingly, the binding of CV-N to PBMC was reduced, but not eliminated, by mannan binding, which implies a second mode of binding between CV-N and the cell surface (Fig. 1K). We assume that distinct populations of differentially labeled cells seen in the flow histograms reflect differences in the amount of labeled protein that binds different subpopulations of leukocytes in the unfractionated PBMC samples.

### Griffithsin bound to PBMC retains its antiviral activity

**Figure 2:**
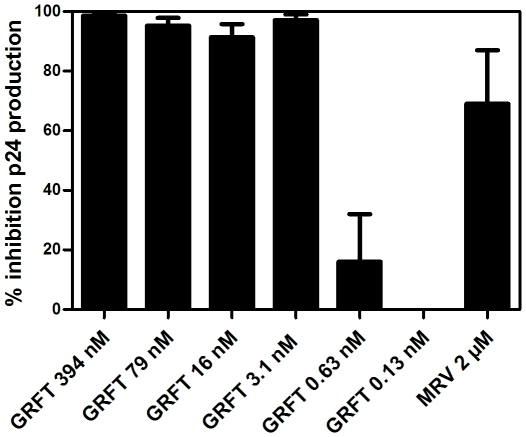
Antiviral activity of GRFT-pretreated PBMC for R5 HIV-1 BaL infection. Freshly isolated PBMC from 3 different donors were pretreated with GRFT (394-0.13 nM) and maraviroc (2 µM) for 24 hrs and then washed and infected with the R5 HIV-1 BaL strain for 9 days. No compounds were added during the infection and viral replication was measured by p24 Ag specific ELISA in the collected supernatants.

When freshly-isolated PBMC were pre-incubated for 24 hrs with GRFT at various concentrations, washed and then infected with HIV-1 R5 strain BaL (without adding new compound), GRFT inhibited viral replication for 9 days of cell culture (Fig. 2). As a control maraviroc (MVR) at 2 µM was included and this also showed anti-HIV activity after 9 days in culture, as this compound is known to bind specifically to the CCR5 receptor. However, lower concentrations of maraviroc (at 0.4 µM) did not retain its antiviral activity in this assay protocol (data not shown).

### High concentrations of GRFT are not cytotoxic to cervico-vaginal cell lines

**Figure 3:**
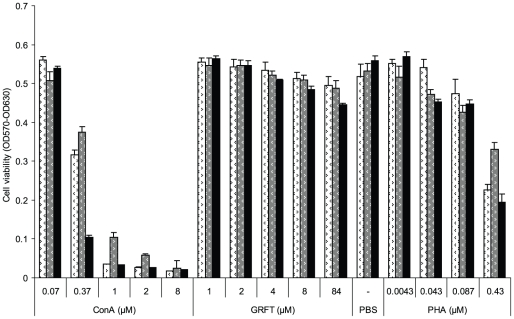
Effect of GRFT on cervico-vaginal cell viability. Cell viability was evaluated in an MTT assay after 72 hours treatment of Ect1/E6E7 (white), End1/E6E7 (grey) and VK2/E6E7 (black) with different test compounds listed on the X-axis together with their concentrations. Values are given as mean ± standard deviation of OD570 – OD630.

An MTT assay was used to assess the effects of GRFT on End1/E6E7, Ec1/E6E7 and VK2/E6E7 cell viability by measuring the metabolic activity of treated cells. In these experiments (Fig. 3) we observed no loss in cell viability after a 3 day exposure of the endocervical and ectocervical cell lines to concentrations of up to 1 mg/ml (84 µM) GRFT, at least 10-times more concentrated than a likely microbicide formulation [23]. High doses of GRFT did, however, slightly reduce viability of the vaginal keratinocyte (VK2) cells. In marked contrast to GRFT, the mannose-specific mitogenic lectin ConA showed clear concentration-dependent cytotoxicity towards all three cell lines. As shown in Fig. 3, treatment with 1 µM ConA killed approximately 94% of the ectocervical cells and vaginal keratinocytes, and 80% of the endocervical cells. PHA, another lectin known to be mitogenic in vivo and in vitro also caused dose-responsive cell death, but to a lesser extent than ConA.

### GRFT does not stimulate cell proliferation

**Figure 4:**
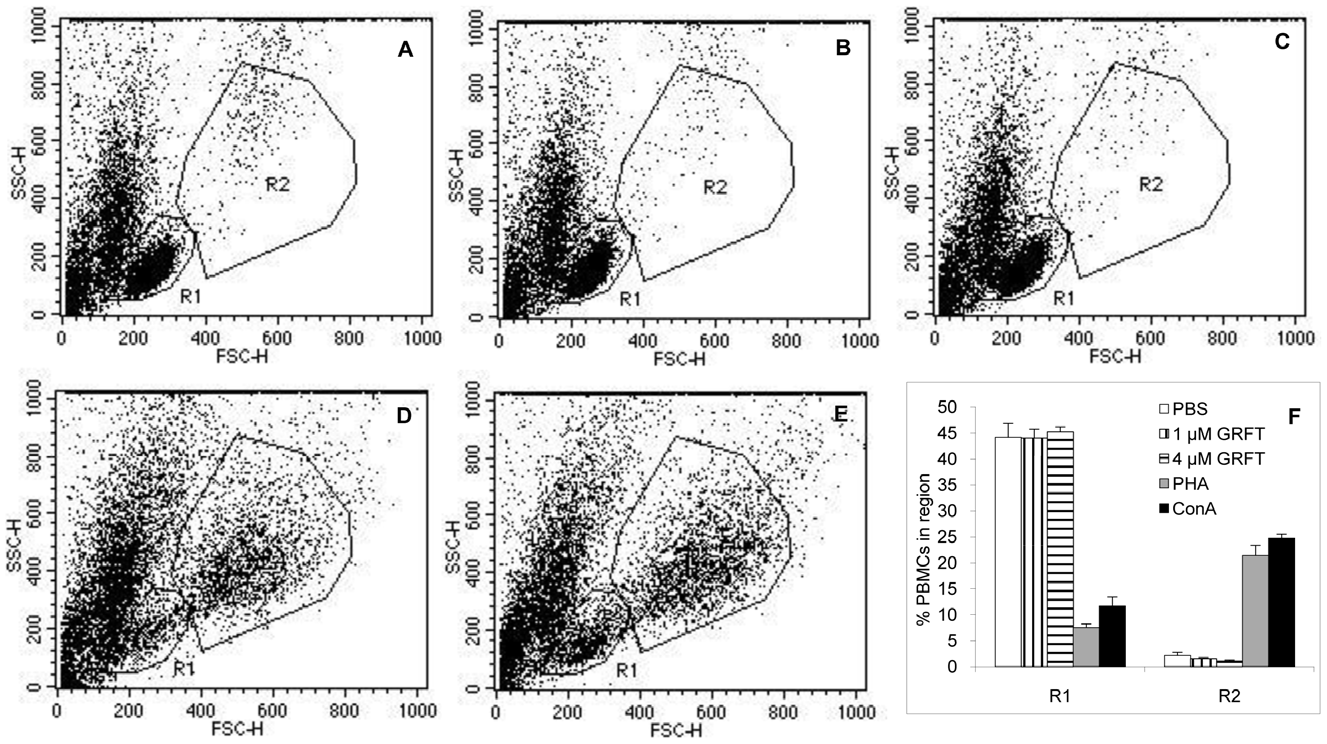
Flow cytometric evaluation of the mitogenic effect of GRFT on PBMC. Cells were analyzed flow-cytometrically after three days treatment with PBS (A), 1 µM GRFT (B), 4 µM GRFT (C), 10 µg/ml PHA (D) and 0.37 µM ConA (E). Typical live PBMC population were gated in region R1 and a subpopulation with increased size and higher SSC was gated in region R2. Quantitation of cells in these regions was plotted in F for each test compound.

**Figure 5:**
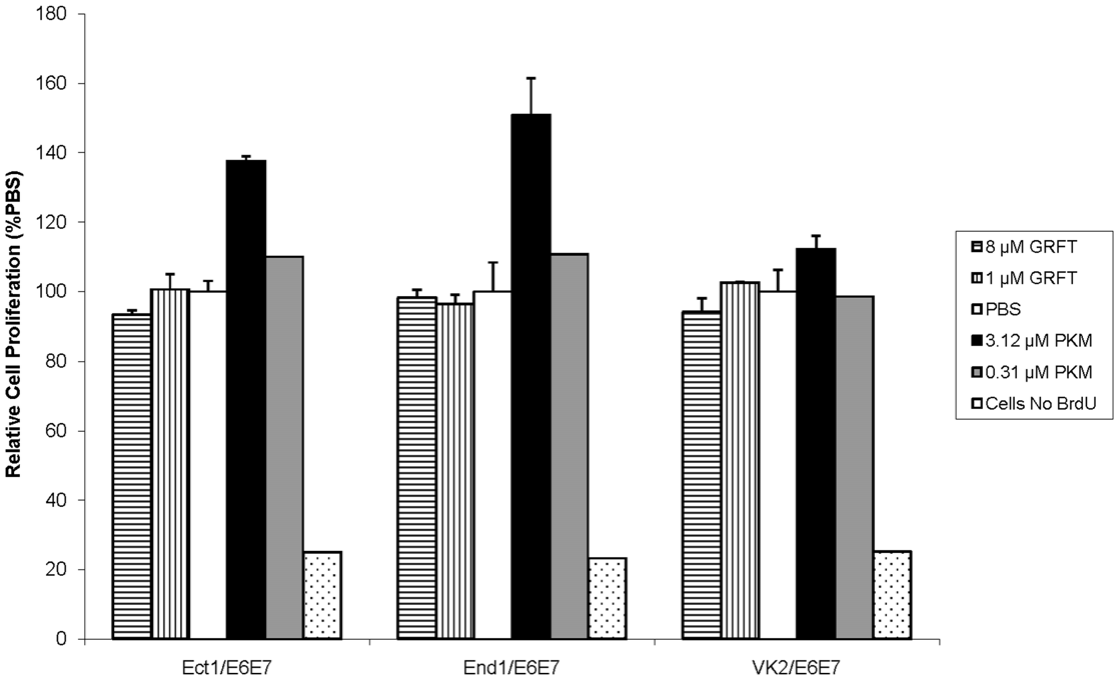
Effect of GRFT on cervico-vaginal cell proliferation as measured by BrdU incorporation. Cell proliferation was measured by the incorporation of BrdU in newly synthesized DNA using a colorimetric assay after treatment of Ect1/E6E7, End1/E6E7 and VK2/E6E7 with GRFT (1 or 8 µM), PKM (3.12 or 0.31 µM) or PBS. Cells without BrdU were assessed in the assay as a negative control according to the manufacturer's intructions. Values are given as mean ± standard deviation of the relative cell proliferation rate derived from BrdU incorporated as a percentage of that found in PBS.

We previously showed that GRFT exhibits no mitogenic stimulatory effect in human PBMC, using incorporation of tritiated thymidine as a marker for cell proliferation [23]. In the present study we investigated the mitogenic activity of GRFT on PBMC by flow cytometry, evaluating changes in size and morphology of cells treated with GRFT in comparison with cells treated with the vehicle (PBS). Cells treated with 1 or 4 µM GRFT had flow cytometry profiles similar to the control cells (Fig. 4A, B and C). In contrast treatment with lectins ConA and PHA at doses (0.37 µM ConA and 10 µg/ml PHA) that do not negatively affect cell viability, resulted in completely different flow cytometric plots, with emergence of a subpopulation composed of larger cells (higher forward scatter FSC) with perceptibly higher side scatter (SSC) values gated in Region R2, as shown in Fig. 4D and E. In addition, a clear decrease in cell number was observed in region R1 after treatment with ConA and PHA, as quantified in Fig. 4F. In Fig. 5, we show that GRFT also does not induce cell proliferation in any of the three cultured human cervical and vaginal epithelial cells. Cell division was assessed by monitoring BrdU incorporation in newly synthesized DNA of actively dividing cervicovaginal cell lines. Treatment with 1 or 8 µM GRFT did not induce proliferation of any of the cell lines since BrdU counts were not elevated in comparison with control cells treated with vehicle alone (PBS). In contrast Pokeweed agglutinin (PKM), a well characterized mitogen, caused concentration-dependent increase in cell division, especially in the cervical cell lines End1/E6E7 and Ect1/E6E7.

### Effects of GRFT treatment on PBMC activation markers

**Figure 6:**
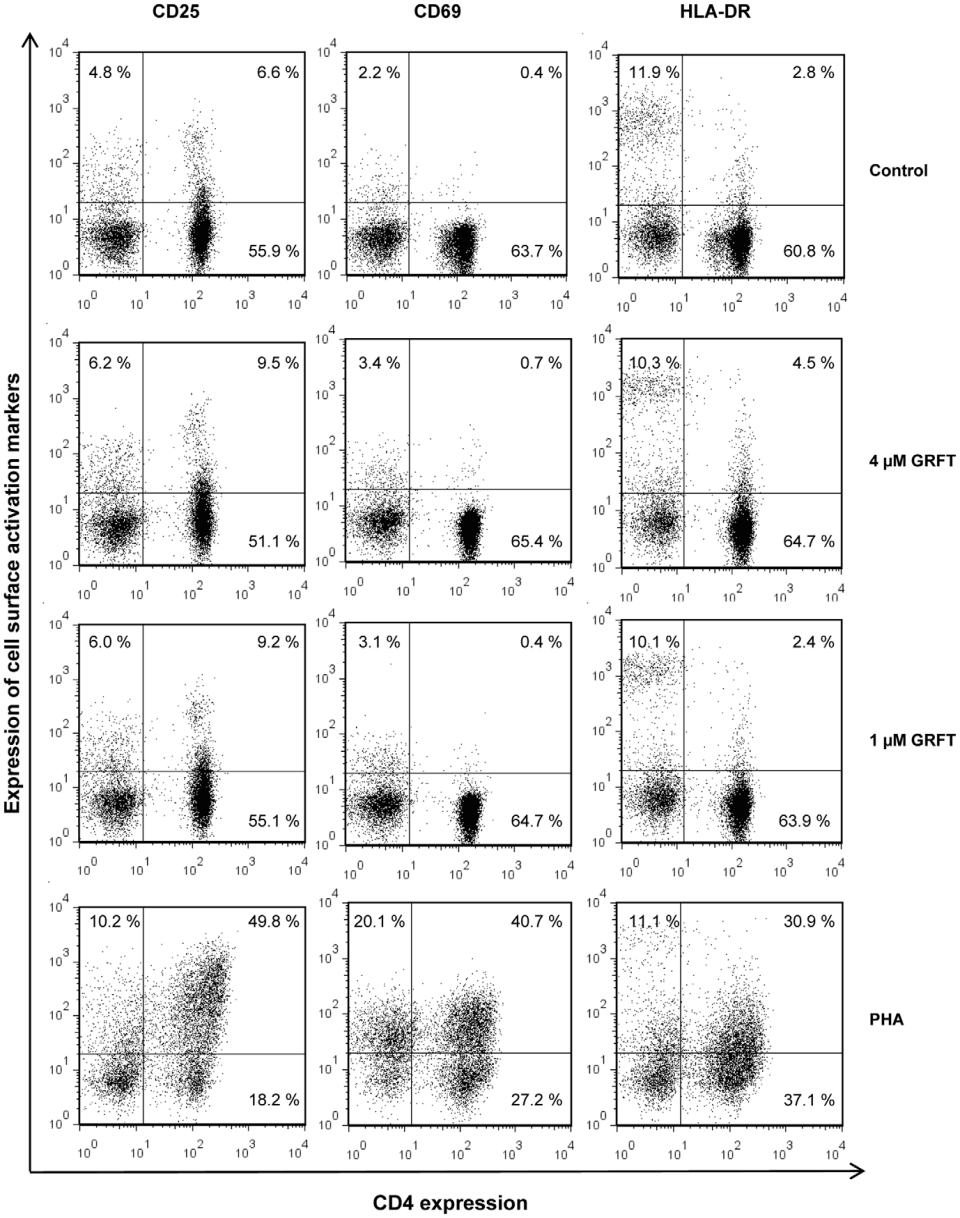
Effect of GRFT on PBMC activation. Cells were incubated for three days in culture medium alone, with 1 µM GRFT, with 4 µM GRFT and with 10 µg/ml PHA and dual stained using FITC-conjugated anti-CD4 mAb in combination with PE-conjugated anti-CD25 (left panel), anti-CD69 (middle panel) and anti-HLA-DR (right panel). The percentages of receptor positive cell populations are indicated in the individual dot plots.

To evaluate the effect of GRFT on cell surface markers of immune activation, we measured expression of the following membrane proteins: (i) CD69, a marker of activated T-lymphocytes, considered an “early” marker of T-cell activation; (ii) CD25, the alpha chain of the IL-2 receptor, upregulated on activated T-cells, B-cells, and some thymocytes and myeloid precursors, considered a “middle” marker of T-cell activation; and (iii) HLA-DR, a component of the type II major histocompatibility (MHC) complex, and a “late” marker of T-cell activation. Activated T-cells are highly susceptible to HIV-1 infection, and hence induction of these markers indicates an undesirable side effect of lectin treatment of PBMC. PBMC were incubated in the presence of the test compounds for 72 hours. The vehicle control was PBS, and positive controls were 10 µg/ml PHA [20], as well as 0.37 µM ConA, chosen as this concentration was not cytotoxic. In PBS treated cells, 1.2% PBMC in average were double positive (CD4^+^/CD25^+^) and a non significant increase of this population was observed after incubation in presence of 1 or 4 µM GRFT (Fig. 6, left panel and Fig. S2A). Treatment with PHA and ConA resulted in an impressive increase in the number of CD4^+^/CD25^+^ cells (Fig. 6, left panel and Fig. S2A). In addition, the numbers of CD4^−^/CD25^+^ cells were elevated when PBMC were cultured in presence of PHA or ConA compared to their PBS and GRFT counterpart (Fig. 6, left panel and data not shown). Similarly, GRFT treatment did not affect significantly the proportion of CD4^+^/CD69^+^ PBMC compared with PBS treatment, whereas ConA and PHA treatment resulted in an increase of more than 10 times in this sub-population (Fig. 6, middle panel and Fig. S2B). The numbers of CD4^−^/CD69^+^ were also elevated after treatment with ConA and PHA (Fig. 6, middle panel, and data not shown). In unstimulated cells, about 15% of CD4 positive PBMC expressed the late activation marker HLA-DR and treatment with GRFT and ConA did not affect this number in a significant fashion (Fig. 6, right panel and Fig. S2C). PHA treatment of cells yielded more than twice the number of CD4^+^/HLA-DR^+^ cells (33.4%) compared with unstimulated PBMC.

### Cytokine and Chemokine Profile of GRFT-incubated PBMC

**Figure 7:**
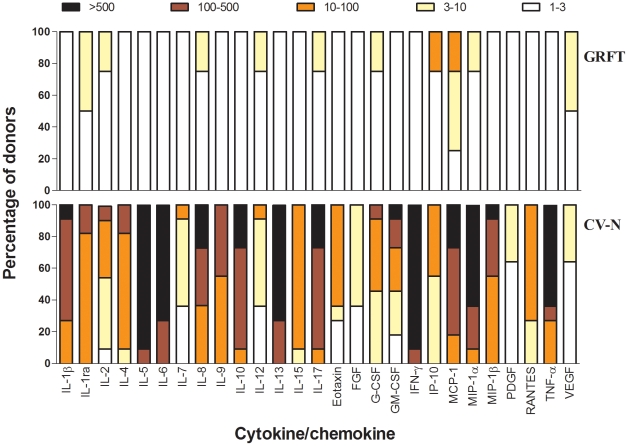
Effect of GRFT on the induction of cytokines/chemokines in PBMC. PBMC from healthy donors were incubated for 72 h with medium only, GRFT or CV-N at 788 nM and 182 nM, respectively. Supernatants were collected and cytokine levels were measured by the Bio-Plex array system. The fold-increase values of the cytokine concentrations in the supernatant of stimulated PBMC with respect to the concentrations in the supernatant of untreated cells were determined from 4 and 11 different donors for GRFT and CV-N, respectively. The fold-increase values are divided in subgroups: 1–3 fold-increase (white squares), 3–10 fold-increase (yellow squares), 10–100 fold-increase (orange squares), 100–500 fold-increase (dark red squares) and >500 fold-increase (black squares). The amount of fold-increase values for each chemokine/cytokine is given as percentage in the total amount of donors (Y-axis).

Activation of PBMC may also be reflected by the production of cytokines and chemokines. To investigate potential activating properties of GRFT more thoroughly, PBMC were cultured in the presence of 10 µg/ml GRFT (788 nM) for 72 h. In the culture supernatant, the concentrations of IL-1α, IL-1ra, IL-2, IL-4, IL-5, IL-6, IL-7, IL-8, IL-9, IL-10, IL-12, IL-13, IL-15, IL-17, eotaxin, FGF, G-CSF, GM-CSF, IFN-γ, IP-10, MCP-1, MIP-1α, MIP-1β, PDGF-BB, RANTES, TNF-α, and VEGF were determined. A detailed overview of the cytokine profiles of GRFT-treated PBMC from multiple blood donors is given in Fig. 7. For reference, we provide previously published data where PBMC with the same donor origins were treated with CV-N at 2 µg/ml (182 nM), lower than the GRFT concentration tested, since higher concentrations of CV-N proved too toxic to PBMC [19]. The concentration of the separate cytokines was compared with that of the untreated PBMC and calculated as a fold increase value. In the previous studies on PBMC treatment with CV-N and MVN, considerable variability in the lectin-induced cytokine profile was observed between the different PBMC donors. Therefore, the fold increase values obtained from the different donors were divided over different ranking groups (*i.e.* 1–3-, 3–10-, 10–100-, 100–500-, and >500-fold increase), and the number in each rank is given as a percentage of the total and indicated by a specific *color* (Fig. 7). Confirming our data in Fig. 6 that GRFT has very little effect on lymphocyte activation markers, we also see minimal alterations in the cytokine and chemokine release for the majority of markers, in most donor PBMC. This profile indicates that GRFT induces significantly less response from PBMC than has been previously reported for CV-N, MVN and ConA [19]. The only chemokine induced weakly by GRFT in the majority of donors (75%) was MCP-1 [19]. Although these studies were performed at a later date than the previously published CV-N and MVN studies, we used exactly the same donor panel, and the assays were performed in the same laboratory (D. Schols), justifying comparison between the experiments.

### Validation of the utility of cultured cervico-vaginal cell lines for detection of off-target effects of GRFT treatment

**Figure 8:**
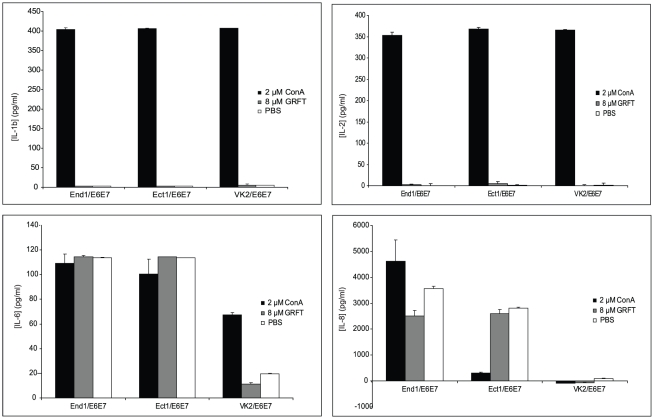
Effect of GRFT on the secretion of selected key cytokines in cervico-vaginal cell lines. Individual ELISA experiments for detection of IL-1b (A), IL-2 (B), IL-6 (C) and IL-8 (D) after 24 hours treatment of cervico-vaginal cells with 2 µM ConA (black), 8 µM GRFT (grey) and PBS (white).

In our previously published studies, we showed that treatment of human cervical explants with a range of GRFT concentrations had no significant effects on expression of a panel of chemokines and cytokines [23]. In the present studies, we used cultured human endocervical, ectocervical and vaginal cell lines for more detailed analysis of potential “off-target” effects of GRFT treatment, since the cell lines are easier to procure than fresh human cervical tissues, and experiments with the cell lines are more easily reproduced without question of variability in genetic background and physiological conditions of the donor. To validate the utility of these cell lines (which are immortalized by induction of papillomavirus E6 and E7 oncogenes) for analysis of any “off-target” effects of GRFT treatment, we wished to confirm that the cell lines behaved similarly to cervical explants after treatment with GRFT, and ConA, which induced inflammatory responses in our PBMC studies. We assessed the secretion of many key mediators of inflammatory responses, including Il-1b, IL-2, IL-6 and IL-8 by all three cervico-vaginal cell lines. ELISA kits specific for these cytokines were used for their detection in End1/E6E7, Ect1/E6E7 and VK2E6E7 cell culture supernatants after 24 h of incubation in presence of the test compounds. After treatment with 8 µM GRFT, the supernatants showed barely detectable levels of IL-1b and IL-2, similar to the cells treated with PBS (Fig. 8A and B). In contrast, all three cell lines produced impressive amounts of these cytokines upon incubation in presence of ConA (Fig. 8A and B). Treatment of cervical epithelial cell lines End1/E6E7and Ect1/E6E7 with GRFT (8 µM), ConA (2 µM) or PBS resulted in similar levels of IL-6 secretion (Fig. 8C), but in VK2/E6E7, IL-6 release was approximately three times higher after ConA treatment compared to PBS, whereas GRFT did not induce the secretion of this cytokine (Fig. 8C). In the case of IL-8, each of the three cell lines showed a distinct pattern of secretion of this cytokine, although no induction was observed after treatment with 8 µM GRFT: VK2/E6E7 showed barely detectable levels of IL-8 regardless of the treatment; IL-8 levels in Ect1/E6E7 were relatively high after PBS and GRFT treatment and these amounts were about ten times reduced after treatment with ConA; GRFT treatment resulted in a slight decrease of IL-8 in End1/E6E7 cell culture supernatants whereas the concentrations of this cytokine were significantly increased following treatment with ConA (Fig. 8D). In addition, GRFT treatment did not alter the production of IL-10, IP-10, MIP-1β and TGF-α in the cervico-vaginal cells studied (data not shown). These data indicated that all 3 cell lines behaved as predicted in response to GRFT and ConA treatment. Since the ectocervical epithelium is an important point of mucosal transmission of HIV-1, we focused on the Ect1/E6E7 cell line to derive a more comprehensive assessment of the effect of GRFT treatment on cytokine production by the cultured ectocervical cells. A multiplexed Luminex assay of the Ect1/E6E7 cell line treated with GRFT confirmed that the Ect1 cells behaved very similarly to human cervical explants (data not shown), and validated our use of these cells in our microarray studies.

### Effect of GRFT Treatment on Gene Expression in Ect1/E6E7 cells

**Figure 9:**
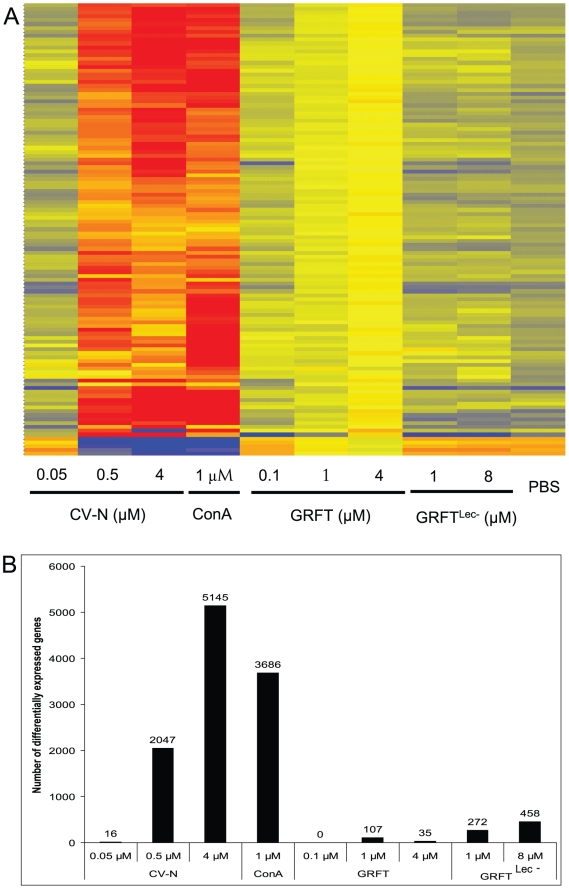
Effect of lectin treatment on gene expression profiles. Cells were treated with CV-N (0.05, 0.5 and 1 µM), ConA (1 µM), GRFT (0.1, 1 and 4 µM), GRFT^Lec-^ (1 and 8 µM) or PBS before RNA isolation, and hybridization with Agilent total human genome microarrays. The heat map (A.) was derived from normalized data using GeneSpring GX 10 software. B. A graphical representation of the total number of genes detected as differentially expressed using Benjamini-Hochberg low stringency correction with a cutoff of 2.0, are reported for CV-N (0.05, 0.5 and 1 µM), ConA (1 µM), GRFT (0.1, 1 and 4 µM), GRFT^Lec-^ (1 and 8 µM) and PBS samples.

Gene expression in Ect1/E6E7 cells in response to treatment with GRFT was compared with vehicle (PBS), GRFT^Lec-^, CV-N and ConA treatments (all qualified with endotoxin levels less than 0.05 EU per milligram). This was studied using a microarray experiment in which the whole human genome was represented by the 41,000 known genes and transcripts [27]. The microarray data were deposited in the Gene Expression Omnibus database, under accession number GSE28584. The heat map shown in Fig. 9A indicates that cells exposed for 24 hours to GRFT^Lec-^ (1 and 8 µM), and low concentrations of GRFT (0.1 µM ) and CV-N (0.05 µM) showed comparable gene expression profiles to those that were incubated in presence of PBS alone. Treatment of Ect1/E6E7 with 1 µM GRFT resulted in minor changes in the expression profile while 4 µM GRFT appeared to alter the expression of many genes but to a much lesser extent compared to CV-N (0.5 and 4 µM) and ConA at 1 µM (Fig. 9). Thus, the microarray studies confirm the data showing that GRFT has substantially lower “off-target” effect in comparison with CV-N and ConA, although there are clearly several genes that are regulated by the carbohydrate binding activity of GRFT (compare with GRFT^Lec-^). As predicted from the heat map (Fig. 9A), no gene was identified as regulated by 0.1 µM GRFT, even when the cutoff was brought to 1.0. However, a first analysis using Benjamini-Hochberg low stringency correction with a cutoff of 2.0 yielded 107 and 35 entries as differentially expressed in samples treated with 1 and 4 µM GRFT, respectively (Fig. 9B). Treatment with CV-N (0.5 and 4 µM) or ConA (1 µM) resulted in regulation of impressive numbers of human genes (Fig. 9B). We then employed stricter criteria by keeping only the positive entries that showed GRFT concentration dependent gene expression. This yielded 2 and 32 genes for 1 and 4 µM GRFT, respectively. The entries which showed an increased gene expression after treatment with 1 µM GRFT included a gene annotated as immunoglobulin-like and fibronectin type III domain containing 1 (IGFN1). The entries that were found to be affected by 4 µM GRFT are summarized in Table S2. Among the 26 genes mapped by the Ingenuity database, there was overrepresentation of genes in the following canonical pathways: NRF2-mediated oxidative stress response (MAF, HMOX1, SOD2), phospholipid degradation, glycerophospholipid metabolism and Endothelin-1 Signaling (HMOX1, WISP2), cAMP mediated signaling (CALML5, PKIB) and acute phase response signaling (HMOX1, SOD2). Furthermore using the Ingenuity software we identified five toxicological functions with an overrepresentation of genes including liver hyperbilirubinemia and steatohepatitis, cardiac arteriopathy, renal and liver necrosis (data not shown). None of these is relevant to mucosal treatment with GRFT. We used quantitative RT-PCR (Q-RT-PCR) to validate the microarray results. Expression of 14 genes was studied using 18S RNA and β-actin mRNA as controls. With the exception of MYCN, all genes studied showed comparable expression levels in both experimental systems including microarrays and Q-PCR (Table S3).

## Discussion

Natural product lectins have received considerable attention as potential antiviral drugs, particularly in the context of prevention of HIV-1 transmission via mucosal surfaces (reviewed recently in [8]). Although the volume of literature supporting their use as antivirals *in vivo* is dwarfed by a comprehensive set of data showing potent *in vitro* antiviral activity, there are reports of impressive *in vivo* efficacy of CV-N in animal models of HIV-1 prevention [17], [18], influenza prevention and treatment [28], and Ebola virus prophylaxis and treatment [29], and of GRFT in prevention of SARS-CoV infection [30]. Despite the myriad of potential prophylactic and therapeutic applications of antiviral lectins, enthusiasm for their development as pharmaceuticals is tempered by a long history of research into natural product lectins, which characterizes many members of this broad class as erythrocyte agglutinins, lymphocyte mitogens, and potentially lethal toxins [10], [11]. The pharmacological basis of natural product lectin toxicity is generally poorly understood, but at a basic mechanistic level is thought to reside in the lectins' capacity for multivalency of binding to cell surface glycans, resulting in cell agglutination and/or cross-linking of cell surface receptor molecules with consequent activation of signaling pathways. Why different lectins that bind identical glycan moieties have quite distinct biological effects *in vitro* remains a paradox that is well illustrated by our data which, show very different *in vitro* activity profiles of four different oligomannose-binding lectins: GRFT, CV-N, MVN [19] and ConA.

Characterization of GRFT is the primary focus of our studies, but understanding the molecular pharmacology and toxicology of this potent antiviral lectin is informed by comparison to CV-N, for which a rich set of *in vitro* and *in vivo* data is available. Both molecules have comparable HIV-1 neutralization activities, with mean IC_80_ values against Clade C viruses of 42.7±4.4 nM for GRFT and 77.0±18.2 for CV-N [15]. Both proteins bind oligomannose glycans, with GRFT targeting terminal mannose residues found on Man_5–9_-GlcNAc_2_ [13] and CV-N specific for the Manα1→2Man linkages found on Man_6–9_-GlcNAc_2_ [16], [31], [32]. They thus share overlapping binding specificities, and should target identical cell surface and viral glycans. If anything, GRFT would be predicted to bind to a larger number of glycan targets than CV-N since it can bind pentamannose structures that lack the α1→2 mannose linkages that CV-N targets [13]. In this context, it is surprising that CV-N appears to bind more promiscuously than GRFT throughout the cervical epithelium and sub-epithelial stroma (Fig. 1A–D, and Fig. S1). Given that GRFT^Lec-^ (the carbohydrate-binding deficient form of GRFT) hardly bound the epithelial sections or cultured cervical and vaginal epithelial cells (Fig. 1), and GRFT binding to cultured epithelial cells was blocked by mannan, we conclude that GRFT's binding activity to the cell surface is exclusively via its carbohydrate binding activity. It is very interesting to note that CV-N binding to the surface of PBMC is not entirely eliminated by blocking its carbohydrate binding sites with mannan (Fig. 1K). This implies that its capacity to bind and induce signaling in cells may not reside entirely in its lectin activities, and supports prior studies with CV-N [21]. Another important result showed that GRFT seems to bind somewhat selectively to terminally differentiated keratinocytes on the epithelial surface, presumably reflecting presence of optimal glycoprotein binding targets on the surface of those cells only in the intact epithelium (Fig. 1C and D; and Fig. S1).

The data presented in Fig. 2 strongly support GRFT's candidacy as a microbicide and antiviral, since they show that GRFT retains antiviral activity even when complexed to the surface of PBMC. This contrasts with many other lectins or compounds we have tested in this assay. The most plausible mechanism that might explain GRFT's retention of HIV-1 entry inhibition activity is that fewer than all six carbohydrate binding sites are occupied when the lectin is docked on cell surface glycoprotein/s, leaving sites available for binding to the viral envelope glycoprotein. The crystal structure of GRFT suggests that the three glycan-binding pockets of each GRFT monomer are on opposite ends of the double prism homodimer [13], suggesting that only one monomer of the homodimer is engaged in binding the cell surface, leaving the “free” monomer competent to bind and cross-link two oligomannose structures on the surface of HIV-1. Since the PBMC were washed extensively 24 hours after treatment and prior to addition of the HIV-1 inoculum, this suggests that cell surface-bound GRFT irreversibly inactivated the inoculum, with no evidence of breakthrough infection at 9 days post infection. This duration of antiviral activity is unprecedented. Conventionally, antiviral potencies of virus-targeted entry inhibitor drugs are measured without washing the cells prior to addition of viruses. In traditional antiviral assays the IC_50_ of GRFT against HIV-1 BaL is 0.2 nM in PBMC, and 0.1 nM against HIV-1 BaL in monocytes/macrophages (data not shown). Remarkably, the IC_50_ for GRFT in the washed PBMC assay (Fig. 2), when the test agent is applied 24 hours prior to cell washing and infection, is 0.78 nM, showing quite exceptional antiviral activity of GRFT. In the same assay we show that activity of CCR5 antagonist maraviroc persists for 24 hrs, although a high concentrations, but GRFT's activity persists substantially longer, a property that may facilitate non-coitally linked administration of microbicides containing GRFT as an active ingredient.

The cardinal rule in development of an anti-HIV-1 microbicide must be “first, do no harm”. Randomized controlled preclinical and clinical studies with detergent-based microbicides such as nonoxynol-9 showed a trend towards evidence of harm, with increased incidence of not only HIV-1 infection, but also HSV-2 and HPV seen in the experimental arms [33], [34], [35], [36], [37]. The microbicides field therefore requires stringent and extensive *in vitro* and *in vivo* safety studies before human trials initiate. Initial studies showed that GRFT was not cytotoxic; had no mitogenic activity; did not induce secretion of chemokine and cytokine-mediators of inflammation in treated cervical explants; and showed a good safety profile in the rabbit vaginal irritation test [23], [24], [25].

The first tissues that GRFT would be exposed to in the human vagina are keratinocytes in the outer surface of the vaginal and cervical epithelia. GRFT would also encounter HIV-1 target cells such as dendritic cells and macrophages resident in the epithelium and submucosal stroma as well as the primary HIV-1 target cells, mucosal CD4+ T-cells. In this work we expanded the safety studies to include analyses of off-target effects derived from GRFT binding cell surface oligomannose glycans on human PBMC as well as characterized ectocervical, endocervical and vaginal keratinocyte cell lines End1/E6E7, Ect1/E6E7 and VK2E6E7, which were originally established from normal human endocervical, ectocervical, and vaginal epithelia, respectively, and immortalized by expression of human papillomavirus 16/E6E7 [38]. The immortalized cell lines were shown to have close resemblance to those of their respective tissues of origin and primary cultures in their morphological and immunocytochemical characteristics and therefore proposed for studies dealing with testing pharmacological agents for intravaginal application [38].

First we evaluated the effects of GRFT on cell viability and we showed that up to 84 µM GRFT did not decrease cell viability in the cervical cell lines End1/E6E7and Ect1/E6E7 whereas VK2/E6E7 was somewhat sensitive to GRFT at high concentrations of 8 µM and above. These results are consistent with the pioneering work of Mori and coworkers in which no significant cellular toxicity was found when a variety of human cell types were treated with GRFT at concentrations of up to 0.783 µM [24]. Since many lectins have been reported to be mitogenic [39], [40], we examined if GRFT would have an effect on the cervico-vaginal cell proliferation. Using a colorimetric assay for detection of BrdU in newly synthesized DNA, we showed that high concentrations of GRFT did not increase the proliferation of either End1/E6E7, Ect1/E6E7 or VK2/E6E7. We then evaluated the concentrations of a panel of cytokines and chemokines in cervico-vaginal cell culture supernatants using ELISA. We found that high concentrations of GRFT had little to no effect on the secretion of cytokines/chemokines studied including Il-1b, IL-2, IL-6, IL-8, IL-10, IP-10, MIP-1b and TGFA in all the three cervico-vaginal cell lines including End1/E6E7, Ect1/E6E7 and VK2/E6E7. Therefore, our results, taken together suggest that GRFT at these concentrations does not alter the secretion of the immune system mediators by cervico-vaginal cells in a significant fashion.

Using the Ect1/E6E7 cell model, we tested GRFT in order to evaluate its effects on gene expression after over night incubation. To our knowledge, this is the first study evaluating the effect of an anti-HIV lectin on gene expression using human genome microarrays. Our data revealed that exposure to GRFT at 0.1 µM did not affect gene expression whereas treatment of Ect1/E6E7 with 1 µM GRFT resulted in minor alteration of the gene expression profile, showing only 2 genes that were considered as significantly upregulated (cutoff of 2.0). Of note our working concentrations were very high. For instance, 1 µM corresponds to about 1,600 times and more than 23,000 times the EC_50_ found for the most resistant and the most sensitive HIV-1 strain, respectively. At higher concentrations of 4 µM GRFT, 32 genes were considered as differentially expressed (cutoff = 2.0). The fold changes found were less or equal to 3.2. Q-RT-PCR evaluation of the expression of selected genes validated the microarray data presented here. It is unclear what is the biological relevance and significance of this level of regulation. Using the same cell line (Ect1/E6E7) Sharkey et al. classified a gene as differentially expressed when the fold change was more than 2.0 and found a total of 444 probe sets that fell in this category after treating the cells 12 h with 10% human seminal plasma [41]. This is in sharp contrast to treatment with 4 µM GRFT, a level at least 1,000 fold greater than the average antiviral EC_50_, which regulated expression of only 32 genes.

In summary, our data provide further evidence that GRFT, an exceptionally potent antiviral lectin, has very minor effects on the molecular physiology of human cells. At this point, the molecular basis for the distinct biological activities of different antiviral lectins is uncharacterized, we propose that the specific spatial arrangement of the carbohydrate binding sites may determine the nature and extent of cross-linking of cell surface glycoproteins. GRFT clearly has superior binding and cross-linking activity with the HIV-1 envelope glycoprotein, which displays dense clusters of oligomannose NLG, but does not induce off-target cellular signaling to the extent that other lectins do. We believe this provides further data in strong support of focused clinical development of HIV-1 microbicides containing GRFT as an active ingredient.

## Materials and Methods

### Lectin reagents

Recombinant GRFT was produced in *Nicotiana benthamiana* plants. Recombinant CV-N was produced in *Escherichia coli*. Methods for expression and purification of both products have been described previously [23], [42]. A synthetic cDNA encoding a lectin activity-deficient mutant of GRFT, termed GRFT^Lec-^, was designed with a conservative amino acid substitution of aspartic acid to asparagine in each of the 3 carbohydrate binding pockets identified in the primary amino acid sequence and crystal structures of GRFT [24], [26]. GRFT^Lec-^ was expressed in *N. benthamiana* and purified exactly as described for GRFT [23]. Proteins were purified to >99% purity, and formulated in phosphate buffered saline (PBS), pH 7.4 at 10 mg/ml protein concentration. Endotoxin was removed from GRFT, GRFT^Lec-^ and CV-N protein samples using Detoxi-Gel endotoxin-removing gel gravity flow columns (Thermo Scientific). Endotoxin levels were measured using the ToxinSensor™ chromogenic LAL endotoxin assay kit from GenScript (Piscataway, NJ). Only products with final Endotoxin readings less than 0.05 Endotoxin Units (EU) per milligram were used in the *in vitro* studies, and all dilutions were performed in Endotoxin-free buffers. GRFT, GRFT^Lec-^ and CV-N were fluorescently-labeled with amine-reactive Alexa Fluor 488 carboxylic acid, succinimidyl ester using a kit from Molecular Probes/Invitrogen, according to the manufacturer's specifications. Control lectins Concanavalin A (ConA), phytohemagglutinin A (PHA) and Pokeweed agglutinin (PKM) were purchased from Sigma.

### Lectin activity measurements using HIV-1 gp120-binding ELISA

Immobilized HIV-1 gp120 (Protein Sciences Corporation) was used to measure the lectin activity of purified GRFT, CV-N and fluorescently labeled conjugates thereof, and to confirm that GRFT^Lec-^ lacked gp120 binding activity. Nunc Maxisorp ELISA plates were coated overnight with 1 µg/ml gp120 (strain IIIB, Protein Sciences) diluted in PBS. The wells were blocked with 5% (w/v) non-fat dry milk in PBS+0.05% Tween (PBS-T; Immunowash, Bio-Rad) and washed before addition of serial dilutions of lectin analyte (GRFT, GRFT^Lec-^, CV-N or Alexa-Fluor 488-labelled conjugates thereof) diluted in 1× PBS for 1 h. After three washes with PBS-T, a primary polyclonal antiserum (rabbit anti-GRFT or CV-N or Alexa-Fluor 488 (Invitrogen), as appropriate) diluted 1∶10,000 in PBS was added for 1 h at room temperature. The wells were again washed before goat anti-rabbit IgG-HRP (Southern Biotech) was added at a 1∶10,000 dilution. Colorimetric values reflecting HRP activity were derived using KPL SureBlue TMB Microwell Peroxidase Substrate, with the reaction stopped by addition of 1 N H_2_SO_4_. The plates were read at 450 nm on a BioTek Synergy HT reader with data collected using Gemini Software. We confirmed that the labeled products retained lectin activities comparable to the unlabeled product by gp120-binding ELISA. We used ELISA with anti-AlexaFluor 488 detection to measure the total amount of label conjugated to GRFT, GRFT^Lec-^ and CV-N, which displayed quantitatively similar labeling efficiency.

### Cervico-vaginal cells lines and human PBMC (PBMC)

End1/E6E7, Ect1/E6E7 and VK2/E6E7 are well characterized immortalized cell lines derived from normal human endocervical, ectocervical and vaginal epithelia, respectively [38]. All 3 cell lines were purchased from the American Type Culture Collection (ATCC, Rockville, MD). The cervico-vaginal cell lines were grown as previously described [38] in keratinocyte serum-free medium (KSFM) supplemented with recombinant human epidermal growth factor (0.1 ng/ml), bovine pituitary extract (50 µg/ml), calcium chloride (0.4 mM) and an antibiotic cocktail composed of penicillin and streptomycin at final concentrations of 100 U/ml and 100 µg/ml, respectively. Reagents were obtained from Invitrogen (SanDiego CA, or from Sigma Chemical Company). Cryopreserved human PBMC used in assays of inflammatory cytokines and chemokines were purchased from SeraCare life Sciences Inc. (Milford, MA) and were immediately cultured for the experiments in RPMI 1640 supplemented with 10% fetal bovine serum (FBS) and the penicillin-streptomycin antibiotic cocktail (to 100 U/ml and 100 µg/ml final concentrations, respectively).

### Microscopical analysis of lectin interaction with human cervical tissues and cultured cervicovaginal cells

Slides with paraffin embedded human cervical tissue sections from a healthy 21 year old female (US Biomax, Inc.) were deparaffinized and rehydrated. Alexa-Fluor 488-labelled proteins of interest were added to the slides and incubated overnight at 4°C in a humidity chamber. The slides were rinsed with PBS twice for 10 minutes and coverslipped using VectaShield Mounting Media with DAPI (Vector Laboratories, Burlingame, CA). For light microscopy, tissue sections were stained with hematoxylin and eosin by standard methods. Ect1/E6E7, End1/E6E7 and VK2/E6E7 cultured cells were seeded onto eight-well Lab-Tek chamber slides (Nalgene Nunc) in duplicate at 10,000 cells per well and allowed to incubate at 37°C with 5% CO_2_. After eight hours the fluorescently labeled proteins of interest were added to the wells and incubated overnight at 37°C with 5% CO_2_. The slides were washed twice for 10 minutes with PBS and coverslipped using VectaShield Mounting Media with DAPI (Vector Laboratories, Burlingame, CA). Slides were visualized using the Axio Observer Z1 microscope with ApoTome assembly (Carl Zeiss, Thornwood, NY).

### Analysis of lectin interaction with PBMC surface molecules by flow cytometry

Human PBMC (SeraCare Life Sciences, MD) were thawed and seeded onto 48-well culture plates (CellTreat, MA) at 2.5×10^5^ cells per well. Three dilutions of each Alexa-Fluor 488-labeled protein were added to the cells for overnight incubation at 37°C with 5% CO_2_. Samples were also prepared with 5 and 10 mg of mannan (Sigma, St. Louis MO) at each protein dilution. All samples were analyzed in duplicate. Following incubation, cells were briefly trypsinized (TrypLE Express, Gibco) and placed in 5 mL polystyrene tubes (BD Falcon, MA). Cells were washed twice with PBS and analyzed using the BD FACSAria (BD Biosciences, NJ) flow cytometer.

### Antiviral activity assay in PBMC

Freshly isolated PBMC were cultured in the presence of GRFT, CV-N and maraviroc for 24 hrs. Then the cells were collected, washed in culture medium, suspended in RPMI medium with 2 ng/ml IL-2 and seeded in a 48-well flat bottom plate (5×10^5^ cells in 450 µl) and 50 µl of the CCR5-tropic clade B HIV-1 BaL stock was added at 100 TCID_50_. The supernatant of each sample was collected after 9 days and viral replication measured by a specific p24 Ag ELISA (Perkin Elmer, Zaventem, Belgium).

### Cell viability assays

Viability of cervico-vaginal cell lines was measured using a colorimetric MTT [3-(4,5-dimethylthiazol-2-yl)2,5-diphenyltetrazolium bromide] assay kit from BIOTIUM Inc. (Hayward, CA) following the manufacturer's instructions. Briefly, 10^4^ cells per well were seeded (96 well plates), the test reagents added and the cultures incubated for three days in a humid environment with 5% CO_2_ at 37°C. Afterwards, 10 µl of MTT solution were added to each well followed by 4 hours incubation at 37°C. Then the medium was gently removed and the insoluble purple formazan product dissolved in DMSO to yield a colored solution which absorbance was read at 570 nm with a background at 630 nm.

### Mitogenicity assays

Proliferation of cervicovaginal cell lines was evaluated by bromodeoxyuridine (BrdU, a thymidine analog) incorporation in newly synthesized DNA using a Cell Proliferation ELISA kit from Roche, according to the manufacturer's instructions. For human PBMC, cells were treated with GRFT for three days and analyzed by flow cytometry for any changes in size and/or morphology using forward scatter (FSC) and side scatter (SSC) with a FACSCalibur (BD, San Jose, CA) counting 10,000 events per sample. Data were acquired and analyzed using CellQuest Pro from BD. ConA (0.37 µM), PHA (10 µg/ml) and PBS were used as controls.

### Evaluation of cellular activition markers

Three day old PBMC were analyzed flow-cytometrically after dual fluorescent staining with anti-mouse antibodies purchased from BD Pharmingen (San Diego, CA). Briefly, cell cultures were transferred from plates to a 5 ml round bottom tubes and washed with PBS containing 5% inactivated FBS (washing solution). After 10 min blocking with purified rat anti-mouse CD16/CD32 (Mouse BD Fc Block), cells were incubated in dark with FITC-conjugated anti-CD4 mAb in combination with PE-conjugated anti-CD25, anti-CD69 or anti HLA-DR mAb for 30 min on ice. Finally PBMC were washed and analyzed with a FACSCalibur (BD, San Jose, CA), counting 10 000 events per sample. Data were acquired and analyzed using CellQuest Pro from BD. ConA (0.37 µM) and PHA (10 µg/ml), and PBS were used as positive and negative controls, respectively.

### Immunoassays for cytokine detection in cultured cervicovaginal cell supernatants

Multiplex immunoassays were carried out on a Bio-Plex instrument (Biorad) using a Milliplex Human Cytokine/Chemokine 42-plex Luminex bead-based assay (Millipore), following the manufacturer's instructions. Each luminex immunoassay was repeated three times. The kit allows simultaneous quantification of human- epidermal growth factor (EGF), eotaxin, fibroblast growth factor-2 (FGF-2), fms-like tyrosine-kinase 3 ligand (Flt-3 ligand), fractalkine, granulocyte colony-stimulating factor (G-CSF), granulocyte-macrophage-CSF (GM-CSF), GRO, interferon- α2(IFN-α2), IFN-γ, interleukin-10 (IL-10), IL-12 (p40), IL-12 (p70), IL-13, IL-15, IL-17, IL-1α, IL-1β, IL-2, IL-3, IL-4, IL-5, IL-6, IL-7, IL-8, IL-9, IL-1 receptor antagonist (IL-1Rα), interferon-inducible protein-10 (IP-10), monocyte chemoattractant protein-1 (MCP-1), MCP-3, macrophage-derived chemokine (MDC), macrophage inflammatory protein-1α (MIP-α), MIP-1β, platelet-derived growth factor-AA (PDGF-AA), PDGF-AB/BB, regulated upon activation normal T-cell expressed and secreted (RANTES), soluble CD40 ligand (sCD40L), soluble IL-2Rα (sIL-2Rα), transforming growth factor α (TGF-α), tumor necrosis factor-α (TNF-α), TNF-β and vascular endothelial growth factor (VEGF). Supernatants collected from Ect1/E6E7 after 24, 48 and 72 hours of culture in presence of test compounds were studied in the multiplex-immuno assays and data were analyzed using the Luminex ×PONENT^R^ software. Individual ELISA experiments were performed on End1/E6E7, Ect1/E6E7 and VK2E6E7 cell culture supernatants collected after 24 hours of incubation in presence of GRFT or controls. ELISA Ready-SET-Go! Kits designed for accurate and precise measurement of human IL-1β, IL-2, IL-6 and IL-10 were purchased from eBioscience, Inc. Quantikine® ELISA kits from R&D Systems, Inc. were used for the detection of IL-8, IP-10 and TGF-α. MIP-1β was quantified using an ELISA kit from Cellsciences. All ELISAs were performed according to the manufacturer's specifications.

### Bio-Plex Cytokine Assay in human PBMC supernatants

PBMC from multiple blood donors [19] were cultured for 72 h in presence of 10 µg/ml GRFT (788 nM). In the culture supernatants, the concentrations of IL-1α, IL-1ra, IL-2, IL-4, IL-5, IL-6, IL-7, IL-8, IL-9, IL-10, IL-12, IL-13, IL-15, IL-17, eotaxin, FGF, G-CSF, GM-CSF, IFN-γ, IP-10, MCP-1, MIP-1α, MIP-1β, PDGF-BB, RANTES, TNF-α, and VEGF were determined by the Bio-Plex 200 system (Bio-Rad) and Bio-Plex Human Cytokine 27-plex assay according to the manufacturer's instructions. Data were generated with Bio-Plex Manager 4.1 software.

### Gene expression analysis in Ect1/E6E7 cells

Cultured Ect1/E6E7 cells were incubated with dilutions of GRFT, GRFT^Lec-^, CV-N, ConA, or vehicle only (PBS, pH 7.4) for 16 hours. The cells were lysed using a Qiagen Qiashredder, and total RNA was extracted using a Qiagen RNeasy Mini Kit. Total RNA was quantified spectrophotometrically and the sample quality was checked on an Agilent 2100 Bioanalyzer (Agilent Technologies, Wilmington, De). 200 ng RNA were used to generate cyanine 3 (Cy3) cRNA with the aid of Low RNA Input Linear Amplification kit, one-color (Agilent Technologies, Wilmington, De) according to the manufacturer's instructions. 1.65 ug of each labeled cRNA sample were fragmented at 60°C for 30 min using an Agilent Gene Expression Hybridization kit (Agilent Technologies, Wilmington, De) followed by hybridization to a whole human genome Agilent oligonucleotide slide containing four high-definition 44 K microarray (Agilent Technologies, Wilmington, De) at 65°C for 17 hours. After hybridization, the slides were washed with Agilent gene expression wash buffers (Agilent Technologies, Wilmington, De) and scanned using an Agilent G2565BA microarray scanner system (Agilent Technologies, Wilmington, De). Then, one-color microarray images were extracted with the Feature Extraction software v 9.5.1 (Agilent Technologies, Wilmington, De) and the raw data imported into GeneSpring GX 10 software (Agilent Technologies, Wilmington, De) for normalization and further analysis to yield a list of differentially expressed genes. Benjammini and Hocheberg correction controlling the false discovery rate FDR [43], [44], [45] was used and we considered only a fold change of at least 2.0 compared to the PBS treated samples to represent a meaningfully altered gene expression [41], [46], [47]. The raw microarray data were uploaded into the Gene Expression Ominbus (GEO) database, a MIAME-compliant database as detailed on the MGED Society Website http://www.mged.org/Workgroups/MIAME.html. The GEO accession number is GSE28584. Further analysis of the regulated genes was carried out using the Ingenuity knowledge database (Ingenuity Systems Inc., Redwood City, CA) yielding putative toxicological functions, and canonical pathways. Quantitative RT-PCR was carried out in order to validate the microarray results. First strand cDNA was reverse transcribed from 250 ng RNA employing a High Capacity RNA-to-cDNA Kit (Applied Biosystems) following the manufacturer's instructions. Optimal amounts of template cDNA were added to a reaction mixture containing 10 µl of 2×TaqMan® Gene Expression Master Mix (Applied Biosystems) and water to 20 µl and this mixture was used to set the PCR reactions in TaqMan® Array Standard 96 well Plates (Applied Biosystems). These plates contain pre-spotted individual TaqMan® Gene Expression Assays for detection of human cysteine-aspartic acid protease (caspase14, CASP14), beta defensin 103A (DEFB103A), immunoglobulin-like and fibronectin type III domain containing 1 (IGFN1), interleukin-1β (IL-1β), IL-2, IL-6, IL-8, IL-10, IL-33, IP-10, *V-Myc* Myelocytomatosis Viral Related Oncogene (MYCN), thyroglobulin (TG ), TGFA, tripartite motif-containing 63 (TRIM63) as well as the house keeping genes 18 S and beta actin (ACTB). Individual probes used in these experiments are described in Table S1. PCR amplification was performed in an 7900HT Fast Real-Time PCR System (AppliedBiosystems) as follows: initial activation step (95°C, 20 min), 40 cycles (95°C, 1 min) and 20 min at 60°C. SDS 2.3 or RQ Manager 1.2 software from Applied Biosystems was used to evaluate the cycle threshold (Ct) for each reaction, which is the cycle number at which 50% maximal amplicon synthesis is achieved. Ratios were derived as proposed elsewhere [48].

### Statistical analyses

Group means and standard deviations were derived from the values obtained in three individual replicates. Statistical significance was analyzed by a one-way analysis of variance (ANOVA) and student's *t*-test unless otherwise stated, using GraphPad software (San Diego, CA). Differences were considered statistically significant if *p*<0.05. For microarray data, the statistical analysis was carried out as described in the *Gene expression analysis* methods above.

## Supporting Information

Figure S1 **Analysis of binding specificity of GRFT in comparison with GRFT^lec-^ and CV-N.** In the first column we depict the identical fluorescence micrographs to Fig. 1, these show binding of AlexaFluor 488-labeled GRFT (B.), GRFT^Lec-^ (C.) and CV-N (D.) to paraffin-embedded cervical tissue sections from a 21-year old female. In (A.) we show a hematoxilin and eosin-stained light micrograph of cervical epithelial tissue, showing the general micro-anatomy of the cervical epithelium. We have provided additional fluorescence micrographs of tissues stained with labeled GRFT (B2 and B3), GRFT^Lec-^ (C2 and C3) and CV-N (D2 and D3).
(TIF)

Figure S2 **Quantitation of the effect of test compounds on the expression of activation markers in CD4 positive PBMC.** Percentages of double positive PBMC are reported for cells after 3 days treatment with PBS, 1 µM GRFT, 4 µM GRFT, 10 µg/ml PHA and 0.37 µM ConA and dual staining using FITC-conjugated anti-CD4 mAb in combination with PE-conjugated anti-CD25 (A), anti-CD69 (B) and anti-HLA-DR (C).
(TIF)

Table S1 Genes used in Q-PCR experiments with corresponding TaqMan assay identities.
(DOC)

Table S2 List and fold changes (FC) of mapped genes differentially expressed after treatment with 4 µM GRFT.
(DOC)

Table S3 Relative expression of selected genes after treatment with GRFT (1 and 4 µM) and 1 µM ConA as assessed by Q-PCR and microarrays (μArrays).
(DOCX)

## References

[1] Wei X, Decker JM, Wang S, Hui H, Kappes JC (2003). Antibody neutralization and escape by HIV-1.. Nature.

[2] Shan M, Klasse PJ, Banerjee K, Dey AK, Iyer SP (2007). HIV-1 gp120 mannoses induce immunosuppressive responses from dendritic cells.. PLoS Pathog.

[3] Doores KJ, Bonomelli C, Harvey DJ, Vasiljevic S, Dwek RA (2010). Envelope glycans of immunodeficiency virions are almost entirely oligomannose antigens.. Proc Natl Acad Sci U S A.

[4] Rudd PM, Dwek RA (1997). Glycosylation: heterogeneity and the 3D structure of proteins.. Crit Rev Biochem Mol Biol.

[5] Scanlan CN, Offer J, Zitzmann N, Dwek RA (2007). Exploiting the defensive sugars of HIV-1 for drug and vaccine design.. Nature.

[6] Knezevic A, Polasek O, Gornik O, Rudan I, Campbell H (2009). Variability, heritability and environmental determinants of human plasma N-glycome.. J Proteome Res.

[7] Chu CS, Ninonuevo MR, Clowers BH, Perkins PD, An HJ (2009). Profile of native N-linked glycan structures from human serum using high performance liquid chromatography on a microfluidic chip and time-of-flight mass spectrometry.. Proteomics.

[8] Francois KO, Balzarini J (2010). Potential of carbohydrate-binding agents as therapeutics against enveloped viruses.. Med Res Rev.

[9] Balzarini J, Van Laethem K, Hatse S, Froeyen M, Peumans W (2005). Carbohydrate-binding agents cause deletions of highly conserved glycosylation sites in HIV GP120: a new therapeutic concept to hit the achilles heel of HIV.. J Biol Chem.

[10] Sharon N, Lis H (2004). History of lectins: from hemagglutinins to biological recognition molecules.. Glycobiology.

[11] Sharon N, Lis H (2007). Lectins.

[12] Matei E, Zheng A, Furey W, Rose J, Aiken C (2010). Anti-HIV activity of defective cyanovirin-N mutants is restored by dimerization.. J Biol Chem.

[13] Moulaei T, Shenoy SR, Giomarelli B, Thomas C, McMahon JB (2010). Monomerization of viral entry inhibitor griffithsin elucidates the relationship between multivalent binding to carbohydrates and anti-HIV activity.. Structure.

[14] Takahashi A, Inokoshi J, Tsunoda M, Suzuki K, Takenaka A (2010). Actinohivin: specific amino acid residues essential for anti-HIV activity.. J Antibiot (Tokyo).

[15] Alexandre KB, Gray ES, Lambson BE, Moore PL, Choge IA (2010). Mannose-rich glycosylation patterns on HIV-1 subtype C gp120 and sensitivity to the lectins, Griffithsin, Cyanovirin-N and Scytovirin.. Virology.

[16] Liu Y, Carroll JR, Holt LA, McMahon J, Giomarelli B (2009). Multivalent interactions with gp120 are required for the anti-HIV activity of Cyanovirin.. Biopolymers.

[17] Tsai CC, Emau P, Jiang Y, Agy MB, Shattock RJ (2004). Cyanovirin-N inhibits AIDS virus infections in vaginal transmission models.. AIDS Res Hum Retroviruses.

[18] Tsai CC, Emau P, Jiang Y, Tian B, Morton WR (2003). Cyanovirin-N gel as a topical microbicide prevents rectal transmission of SHIV89.6P in macaques.. AIDS Res Hum Retroviruses.

[19] Huskens D, Ferir G, Vermeire K, Kehr JC, Balzarini J (2010). Microvirin, a novel alpha(1,2)-mannose-specific lectin isolated from Microcystis aeruginosa, has anti-HIV-1 activity comparable with that of cyanovirin-N but a much higher safety profile.. J Biol Chem.

[20] Huskens D, Vermeire K, Vandemeulebroucke E, Balzarini J, Schols D (2008). Safety concerns for the potential use of cyanovirin-N as a microbicidal anti-HIV agent.. Int J Biochem Cell Biol.

[21] Balzarini J, Van Laethem K, Peumans WJ, Van Damme EJ, Bolmstedt A (2006). Mutational pathways, resistance profile, and side effects of cyanovirin relative to human immunodeficiency virus type 1 strains with N-glycan deletions in their gp120 envelopes.. J Virol.

[22] Buffa V, Stieh D, Mamhood N, Hu Q, Fletcher P (2009). Cyanovirin-N potently inhibits human immunodeficiency virus type 1 infection in cellular and cervical explant models.. J Gen Virol.

[23] O'Keefe BR, Vojdani F, Buffa V, Shattock RJ, Montefiori DC (2009). Scaleable manufacture of HIV-1 entry inhibitor griffithsin and validation of its safety and efficacy as a topical microbicide component.. Proc Natl Acad Sci U S A.

[24] Mori T, O'Keefe BR, Sowder RC, Bringans S, Gardella R (2005). Isolation and characterization of griffithsin, a novel HIV-inactivating protein, from the red alga Griffithsia sp.. J Biol Chem.

[25] Emau P, Tian B, O'Keefe BR, Mori T, McMahon JB (2007). Griffithsin, a potent HIV entry inhibitor, is an excellent candidate for anti-HIV microbicide.. J Med Primatol.

[26] Ziolkowska NE, O'Keefe BR, Mori T, Zhu C, Giomarelli B (2006). Domain-swapped structure of the potent antiviral protein griffithsin and its mode of carbohydrate binding.. Structure.

[27] Ibanez de Caceres I, Cortes-Sempere M, Moratilla C, Machado-Pinilla R, Rodriguez-Fanjul V (2010). IGFBP-3 hypermethylation-derived deficiency mediates cisplatin resistance in non-small-cell lung cancer.. Oncogene.

[28] Smee DF, Bailey KW, Wong MH, O'Keefe BR, Gustafson KR (2008). Treatment of influenza A (H1N1) virus infections in mice and ferrets with cyanovirin-N.. Antiviral Res.

[29] Barrientos LG, O'Keefe BR, Bray M, Sanchez A, Gronenborn AM (2003). Cyanovirin-N binds to the viral surface glycoprotein, GP1,2 and inhibits infectivity of Ebola virus.. Antiviral Res.

[30] O'Keefe BR, Giomarelli B, Barnard DL, Shenoy SR, Chan PK (2010). Broad-spectrum in vitro activity and in vivo efficacy of the antiviral protein griffithsin against emerging viruses of the family Coronaviridae.. J Virol.

[31] Barrientos LG, Matei E, Lasala F, Delgado R, Gronenborn AM (2006). Dissecting carbohydrate-Cyanovirin-N binding by structure-guided mutagenesis: functional implications for viral entry inhibition.. Protein Eng Des Sel.

[32] Yang F, Bewley CA, Louis JM, Gustafson KR, Boyd MR (1999). Crystal structure of cyanovirin-N, a potent HIV-inactivating protein, shows unexpected domain swapping.. J Mol Biol.

[33] Roberts JN, Buck CB, Thompson CD, Kines R, Bernardo M (2007). Genital transmission of HPV in a mouse model is potentiated by nonoxynol-9 and inhibited by carrageenan.. Nat Med.

[34] Beer BE, Doncel GF, Krebs FC, Shattock RJ, Fletcher PS (2006). In vitro preclinical testing of nonoxynol-9 as potential anti-human immunodeficiency virus microbicide: a retrospective analysis of results from five laboratories.. Antimicrob Agents Chemother.

[35] Stafford MK, Ward H, Flanagan A, Rosenstein IJ, Taylor-Robinson D (1998). Safety study of nonoxynol-9 as a vaginal microbicide: evidence of adverse effects.. J Acquir Immune Defic Syndr Hum Retrovirol.

[36] Galen BT, Martin AP, Hazrati E, Garin A, Guzman E (2007). A comprehensive murine model to evaluate topical vaginal microbicides: mucosal inflammation and susceptibility to genital herpes as surrogate markers of safety.. J Infect Dis.

[37] Marais D, Carrara H, Kay P, Ramjee G, Allan B (2006). The impact of the use of COL-1492, a nonoxynol-9 vaginal gel, on the presence of cervical human papillomavirus in female sex workers.. Virus Res.

[38] Fichorova RN, Rheinwald JG, Anderson DJ (1997). Generation of papillomavirus-immortalized cell lines from normal human ectocervical, endocervical, and vaginal epithelium that maintain expression of tissue-specific differentiation proteins.. Biol Reprod.

[39] Balzarini J (2006). Inhibition of HIV entry by carbohydrate-binding proteins.. Antiviral Res.

[40] Van Damme EJ, Barre A, Verhaert P, Rouge P, Peumans WJ (1996). Molecular cloning of the mitogenic mannose/maltose-specific rhizome lectin from Calystegia sepium.. FEBS Lett.

[41] Sharkey DJ, Macpherson AM, Tremellen KP, Robertson SA (2007). Seminal plasma differentially regulates inflammatory cytokine gene expression in human cervical and vaginal epithelial cells.. Mol Hum Reprod.

[42] Mori T, Gustafson KR, Pannell LK, Shoemaker RH, Wu L (1998). Recombinant production of cyanovirin-N, a potent human immunodeficiency virus-inactivating protein derived from a cultured cyanobacterium.. Protein Expr Purif.

[43] Benjamini Y, Drai D, Elmer G, Kafkafi N, Golani I (2001). Controlling the false discovery rate in behavior genetics research.. Behav Brain Res.

[44] Reiner A, Yekutieli D, Benjamini Y (2003). Identifying differentially expressed genes using false discovery rate controlling procedures.. Bioinformatics.

[45] Breitling R (2006). Biological microarray interpretation: the rules of engagement.. Biochim Biophys Acta.

[46] Booij JC, ten Brink JB, Swagemakers SM, Verkerk AJ, Essing AH (2010). A new strategy to identify and annotate human RPE-specific gene expression.. PLoS One.

[47] Li Z, Liu B, Maminishkis A, Mahesh SP, Yeh S (2008). Gene expression profiling in autoimmune noninfectious uveitis disease.. J Immunol.

[48] Livak KJ, Schmittgen TD (2001). Analysis of relative gene expression data using real-time quantitative PCR and the 2(-Delta Delta C(T)) Method.. Methods.

