# Supplementary figures and images for: Investigation of Griffithsin's Interactions with Human Cells Confirms Its Outstanding Safety and Efficacy Profile as a Microbicide Candidate
Source: PLoS One. 2011 Aug 2;6(8):e22635. doi: 10.1371/journal.pone.0022635 (PMC3149051; doi:10.1371/journal.pone.0022635)

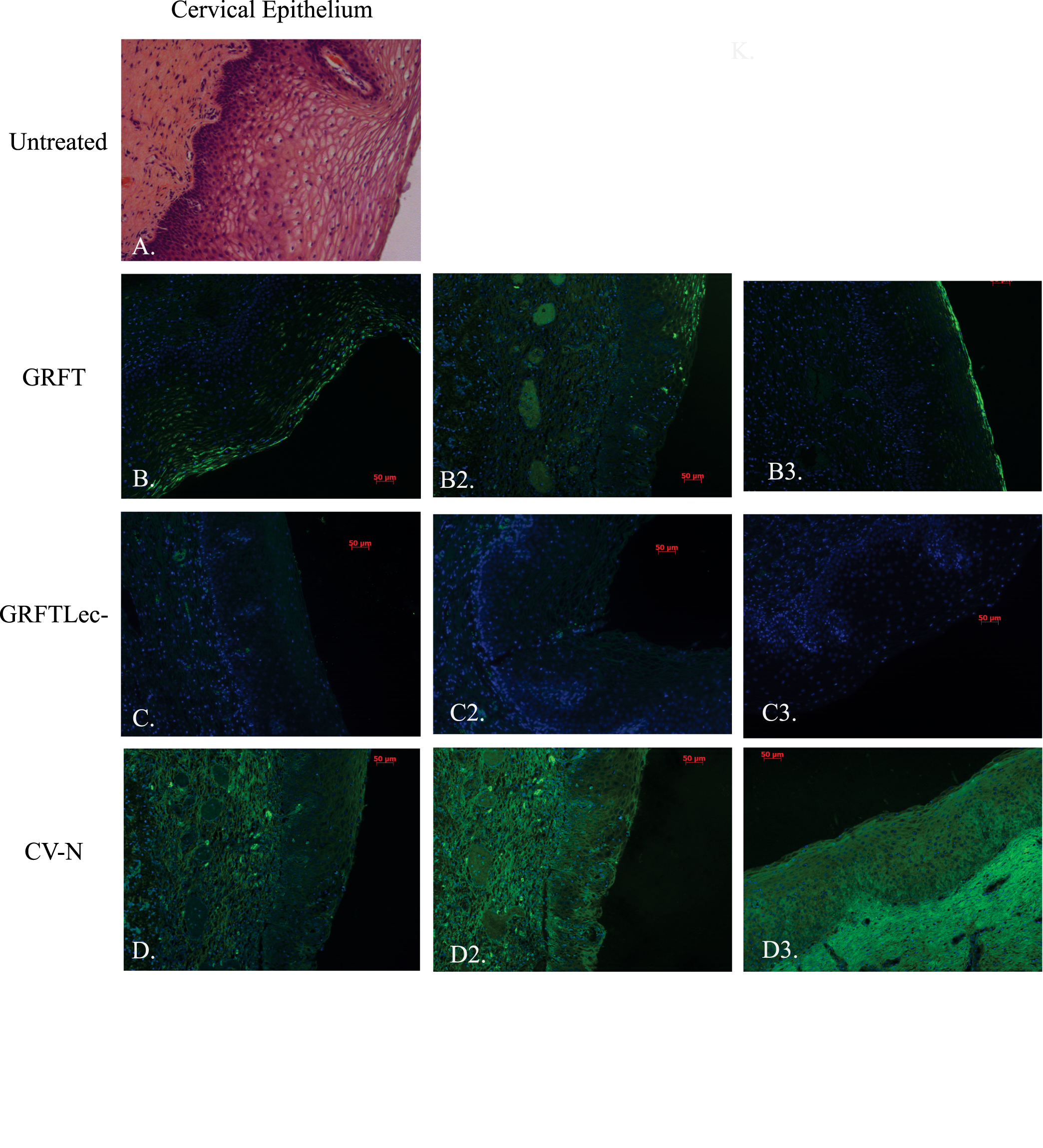

Supplement: Figure S1 — Analysis of binding specificity of GRFT in comparison with GRFTlec- and CV-N. In the first column we depict the identical fluorescence micrographs to Fig. 1, these show binding of AlexaFluor 488-labeled GRFT (B.), GRFTLec- (C.) and CV-N (D.) to paraffin-embedded cervical tissue sections from a 21-year old female. In (A.) we show a hematoxilin and eosin-stained light micrograph of cervical epithelial tissue, showing the general micro-anatomy of the cervical epithelium. We have provided additional fluorescence micrographs of tissues stained with labeled GRFT (B2 and B3), GRFTLec- (C2 and C3) and CV-N (D2 and D3). (TIF) [file pone.0022635.s001.tif]
