# Supplementary material for: Investigation of Griffithsin's Interactions with Human Cells Confirms Its Outstanding Safety and Efficacy Profile as a Microbicide Candidate
Source: PLoS One. 2011 Aug 2;6(8):e22635. doi: 10.1371/journal.pone.0022635 (PMC3149051; doi:10.1371/journal.pone.0022635)
Supplement: Table S1 — Genes used in Q-PCR experiments with corresponding TaqMan assay identities. (DOC) [file pone.0022635.s003.doc]

**Table S1**. Genes used in Q-PCR experiments with corresponding TaqMan assay identities

|  | Assay ID | Amplicon length | Fluorophore |
| --- | --- | --- | --- |
| 18S | Hs99999901_s1 | 187 | FAM |
| ACTB | Hs03023943_g1 | 96 | FAM |
| CASP14 | Hs00201637_m1 | 88 | FAM |
| DEFB103A | Hs00218678_m1 | 93 | FAM |
| IGFN1 | Hs00297580_m1 | 81 | FAM |
| IL-1β | Hs01555410_m1 | 91 | FAM |
| IL-10 | Hs99999035_m1 | 86 | FAM |
| IL2 | [Hs00914135_m1](https://products.appliedbiosystems.com:443/ab/en/US/adirect/ab?cmd=ABAssayDetailDisplay&assayID=Hs00914135_m1&Fs=y&SearchRequest.Common.PageNumber=1&assayType=ge&chkBatchQueryText=false&srchType=keyword&searchValue=Hs00914135_m1&searchBy=all&msgType=ABGEKeywordResults) | 104 | FAM |
| IL-33 | Hs00369211_m1 | 98 | FAM |
| Il-6 | Hs00174131_m1 | 95 | FAM |
| IL-8 | Hs01553824_g1 | 91 | FAM |
| IP-10 | Hs00171042_m1 | 98 | FAM |
| MYCN | Hs00232074_m1 | 81 | FAM |
| TG | Hs00794359_m1 | 81 | FAM |
| TGFA | Hs00177401_m1 | 95 | FAM |
| TRIM63 | Hs00822397_m1 | 94 | FAM |
