# Supplementary material for: Investigation of Griffithsin's Interactions with Human Cells Confirms Its Outstanding Safety and Efficacy Profile as a Microbicide Candidate
Source: PLoS One. 2011 Aug 2;6(8):e22635. doi: 10.1371/journal.pone.0022635 (PMC3149051; doi:10.1371/journal.pone.0022635)
Supplement: Table S2 — List and fold changes (FC) of mapped genes differentially expressed after treatment with 4 µM GRFT. (DOC) [file pone.0022635.s004.doc]

**Table S2**. List and fold changes (FC) of mapped genes differentially expressed after treatment with 4 μM GRFT

| Agilent Probe number | Entrez Gene Name | Symbol | FC |
| --- | --- | --- | --- |
| A_24_P235429 | ATP-binding cassette, sub-family A (ABC1), member 1 | ABCA1 | 2.2 |
| A_32_P154053 | ATG9 autophagy related 9 homolog B (S. Cerevisiae) | ATG9B | 2.5 |
| A_23_P146146 | Atpase, H+ transporting, lysosomal 38kda, V0 subunit d2 | ATP6V0D2 | 2.2 |
| A_24_P329795 | Chromosome 10 open reading frame 10 | C10ORF10 | 2.8 |
| A_23_P124095 | Calmodulin-like 5 | CALML5 | 2.0 |
| A_23_P78958 | Calcyphosine | CAPS | 2.0 |
| A_24_P382319 | Carcinoembryonic antigen-related cell adhesion molecule 1 (biliary glycoprotein) | CEACAM1 | 2.2 |
| A_23_P137665 | Chitinase 3-like 1 (cartilage glycoprotein-39) | CHI3L1 | 2.3 |
| A_23_P27473 | Cornifelin | CNFN | 2.1 |
| A_23_P169017 | Defensin, beta 103A | DEFB103A | 2.9 |
| A_23_P120883 | Heme oxygenase (decycling) 1 | HMOX1 | 2.4 |
| A_32_P425998 | Immunoglobulin-like and fibronectin type III domain containing 1 | IGFN1 | 3.2 |
| A_23_P31945 | Interleukin 33 | IL33 | 2.0 |
| A_32_P170454 | Hypothetical protein LOC283454 | LOC283454 | 2.1 |
| A_23_P397376 | V-maf musculoaponeurotic fibrosarcoma oncogene homolog (avian) | MAF | 2.6 |
| A_23_P145529 | Protein kinase (camp-dependent, catalytic) inhibitor beta | PKIB | 2.1 |
| A_24_P280274 | S100 calcium binding protein A7A | S100A7A | 2.0 |
| A_32_P187571 | Sodium channel, voltage-gated, type II, beta | SCN2B | 2.2 |
| A_32_P83098 | Sodium channel, nonvoltage-gated 1, beta | SCNN1B | 2.3 |
| A_24_P935819 | Superoxide dismutase 2, mitochondrial | SOD2 | 2.2 |
| A_24_P8371 | Spinster homolog 2 (Drosophila) | SPNS2 (includes EG:124976) | 2.2 |
| A_24_P392201 | TBC1 domain family, member 10C | TBC1D10C | 2.2 |
| A_23_P32454 | Thyroglobulin | TG | 2.1 |
| A_32_P66035 | Transmembrane protein 86A | TMEM86A | 2.0 |
| A_23_P114983 | Tripartite motif-containing 63 | TRIM63 | 3.0 |
| A_23_P102611 | WNT1 inducible signaling pathway protein 2 | WISP2 | 2.0 |
