# Supplementary material for: Investigation of Griffithsin's Interactions with Human Cells Confirms Its Outstanding Safety and Efficacy Profile as a Microbicide Candidate
Source: PLoS One. 2011 Aug 2;6(8):e22635. doi: 10.1371/journal.pone.0022635 (PMC3149051; doi:10.1371/journal.pone.0022635)
Supplement: Table S3 — Relative expression of selected genes after treatment with GRFT (1 and 4 µM) and 1 µM ConA as assessed by Q-PCR and microarrays (μArrays). (DOCX) [file pone.0022635.s005.docx]

**Table S3**. Relative expression of selected genes after treatment with GRFT (1 and 4 μM) and 1 μM ConA as assessed by Q-PCR and microarrays (μArrays)

| Gene symbol | 1 μM GRFT | | 4 μM GRFT | | 1 μM ConA | |
| --- | --- | --- | --- | --- | --- | --- |
|  | Q-PCR | μArrays | Q-PCR | μArrays | Q-PCR | μArrays |
| CASP14 | 1.72 ± 0.24 | 1.00 | 1.95 ± 0.41 | 1.75 | 4.49 ± 0.63 | 5.17 |
| DEFB103A | 2.94 ± 0.44 | 1.97 | 3.82 ± 0.89 | 2.88 | 1.79 ± 0.56 | 1.79 |
| IGFN1 | 4.78 ± 3.63 | 2.32 | 6.04 ± 3.36 | 3.24 | 1.24 ± 1.11 | 1.00 |
| IL10 | 0.95 ± 0.08 | 1.00 | 0.92 ± 0.07 | 1.00 | 2.62 ± 1.95 | 1.00 |
| IL1B | 0.89 ± 0.18 | 1.00 | 0.69 ± 0.11 | 0.77 | 0.29 ± 0.04 | 0.47 |
| IL-2 | 1.08 ± 0.11 | 1.00 | 0.92 ± 0.07 | 1.00 | 0.91 ± 0.31 | 1.00 |
| IL33 | 2.70 ± 0.77 | 1.43 | 2.66 ± 0.37 | 2.01 | 0.77 ± 0.65 | 0.75 |
| IL6 | 0.97 ± 0.23 | 1.00 | 0.92 ± 0.24 | 1.00 | 0.87 ± 0.24 | 1.13 |
| IL8 | 1.46 ± 0.43 | 1.00 | 1.47 ± 0.57 | 1.00 | 0.91 ± 0.25 | 1.00 |
| IP10 | 0.89 ± 0.18 | 1.00 | 1.18 ± 0.17 | 1.00 | 1.12 ± 0.31 | 1.00 |
| MYCN | 1.08 ± 0.11 | 12.0 | 7.04 ± 4.95 | 1.00 | 10.32 ± 2.05 | 1.00 |
| TG | 2.27 ± 0.54 | 1.00 | 5.15 ± 3.92 | 2.14 | 27.97 ± 11.77 | 14.82 |
| TGFA | 0.91 ± 0.24 | 1.0 | 0.96 ± 0.30 | 1.0 | 0.55 ± 0.19 | 0.56 |
| TRIM63 | 2.34 ± 0.08 | 1.97 | 4.21 ± 0.68 | 3.04 | 4.42 ± 0.43 | 5.99 |
